# *In vivo* Sarcomere Lengths and Sarcomere Elongations Are Not Uniform across an Intact Muscle

**DOI:** 10.3389/fphys.2016.00187

**Published:** 2016-05-25

**Authors:** Eng Kuan Moo, Rafael Fortuna, Scott C. Sibole, Ziad Abusara, Walter Herzog

**Affiliations:** Human Performance Laboratory, Faculty of Kinesiology, University of CalgaryCalgary, AB, Canada

**Keywords:** sarcomere length, non-uniformity, second harmonic generation imaging, myosin band, *in vivo*, passive lengthening

## Abstract

Sarcomere lengths have been a crucial outcome measure for understanding and explaining basic muscle properties and muscle function. Sarcomere lengths for a given muscle are typically measured at a single spot, often in the mid-belly of the muscle, and at a given muscle length. It is then assumed implicitly that the sarcomere length measured at this single spot represents the sarcomere lengths at other locations within the muscle, and force-length, force-velocity, and power-velocity properties of muscles are often implied based on these single sarcomere length measurements. Although, intuitively appealing, this assumption is yet to be supported by systematic evidence. The objective of this study was to measure sarcomere lengths at defined locations along and across an intact muscle, at different muscle lengths. Using second harmonic generation (SHG) imaging technique, sarcomere patterns in passive mouse tibialis anterior (TA) were imaged in a non-contact manner at five selected locations (“proximal,” “distal,” “middle,” “medial,” and “lateral” TA sites) and at three different lengths encompassing the anatomical range of motion of the TA. We showed that sarcomere lengths varied substantially within small regions of the muscle and also for different sites across the entire TA. Also, sarcomere elongations with muscle lengthening were non-uniform across the muscle, with the highest sarcomere stretches occurring near the myotendinous junction. We conclude that muscle mechanics derived from sarcomere length measured from a small region of a muscle may not well-represent the sarcomere length and associated functional properties of the entire muscle.

## Introduction

Sarcomeres are the basic force producing units of muscles. Under a light microscope, muscle fibers have alternating black and white bands due to the contractile filaments myosin (anisotropic band, A-band) and actin (isotropic band, I-band) that make up sarcomeres. Sarcomere patterns are useful indicators of myofibril integrity and have been used in diagnosing muscle pathologies (Plotnikov et al., 2008; Ralston et al., 2008). In addition, the amount of force and power a muscle can generate depends on the sarcomere lengths (Gordon et al., 1966; Lutz and Rome, 1994; Burkholder and Lieber, 1998).

Sarcomere lengths have been a crucial outcome measure for understanding and explaining basic muscle properties(Huxley and Peachey, 1961; Hill, 1953; Morgan, 1994) and muscle function within the constraints of animal bodies (Cutts, 1988; Lutz and Rome, 1994). Sarcomere length can be measured readily from muscle micrographs generated by light microscopy (Huxley and Peachey, 1961; Telley et al., 2006; Infantolino et al., 2010) and electron microscopy (Goulding et al., 1997), or from the diffraction pattern resulting from shining a laser beam through a muscle (ter Keurs et al., 1978). For light or electron microscopy, muscle cells need to be isolated through mechanical or enzymatic means (Huxley and Peachey, 1961; Goulding et al., 1997; Telley et al., 2006; Infantolino et al., 2010). Although, laser diffraction allows measurement of sarcomere length *in vivo* (Takahashi et al., 2007), a small muscle fascicle must be locally dissociated from the whole muscle for full penetration of the laser light. As a result, all of the aforementioned approaches involve a high degree of invasiveness. Recent advances in non-linear microscopy make visualization of sarcomeres in living muscles possible through second harmonic generation (SHG) imaging (Campagnola and Loew, 2003; Plotnikov et al., 2006). Sarcomeres were successfully imaged *in vivo* using a micrometer-sized endoscope (Llewellyn et al., 2008; Cromie et al., 2013). However, this approach requires the insertion of the micro-endoscope into the muscle and it remains unclear what effect such a mechanical perturbation might have on the resulting sarcomere length. Therefore, an improvement to existing techniques involves the examination of sarcomere lengths that involves no contact with the muscle.

Sarcomere length for a given muscle is typically measured at a single spot, often in the mid-belly of the muscle, and at a given muscle length (e.g., Cutts, 1988). It is then assumed implicitly that the sarcomere length measured at this single spot represents the sarcomere lengths at other locations within the muscle, and force-length, force-velocity, and power-velocity properties of muscles are often implied based on these single sarcomere length measurements (Rack and Westbury, 1969; Lutz and Rome, 1994; Burkholder and Lieber, 2001; Vaz et al., 2012). However, it has been shown in single fibers (Huxley and Peachey, 1961; Infantolino et al., 2010), and whole muscles (Llewellyn et al., 2008) that sarcomere lengths can be, and typically are quite variable. However, sarcomere length variability within a muscle has not been systematically analyzed. Furthermore, it has been tacitly assumed that when muscles change length, all fibers in the muscle, independent of their initial length, change length in such a manner that sarcomere lengths remain uniform across the entire muscle (Cutts, 1988), an intuitively appealing assumption, but one that has no systematic scientific support.

Therefore, the purpose of this study was to measure sarcomere lengths at defined locations along and across an intact muscle, at different muscle lengths. Sarcomere length variations were quantified among approximately 240–320 sarcomeres at a given location, for five different locations within the muscle, and three different lengths encompassing the anatomical range of motion of the mouse tibialis anterior (TA). All measurements were performed in passive muscle. We hypothesized that sarcomere lengths differ locally, as has been shown in single fibers and entire muscles before. In addition, based on the implicit assumption used in previous studies, we hypothesized that mean sarcomere lengths were similar at different locations of the muscle and that sarcomere length changes were also uniform across the different locations.

## Materials and methods

### Animal preparation

All aspect of animal care and experimental procedures were carried out in accordance with the guidelines of the Canadian Council on Animal Care and were approved by the University of Calgary Life Sciences Animal Research and Ethics Committee. Ten to twelve week-old male C57BL6 mice (*n* = 5) were anesthetized using a 1–3% isoflurane/oxygen mixture that was delivered through mask ventilation at a flow rate of 0.6 l/min. The state of anesthesia was monitored through foot pinch of the contralateral limb. The left hind limb was skinned. The fascia over the tibialis anterior (TA) muscle was removed. Then, the left knee of the mouse was fixed by a custom-made clamp, while the left foot was pinned to a movable base that allowed adjustment of the ankle angle. The Achilles tendon of the left hind limb was cut to allow for a full range of motion of the ankle without resistance from the triceps surae muscles. Throughout the experiment, the left TA muscle was immersed in a phosphate buffered saline (P5368, Sigma Aldrich, Ontario, Canada) with a pH of 7.4 in a custom-made water chamber and was kept at room temperature (~21°C) to keep the muscle hydrated and to allow for water-immersion imaging. After the imaging protocols, the mice were sacrificed by a barbiturate overdose using 0.5 ml Euthanyl (pentobarbital sodium, Biomeda-MTC pharmaceuticals, Cambridge, Ontario, Canada).

### *In vivo* imaging of muscle

**Figure 1:**
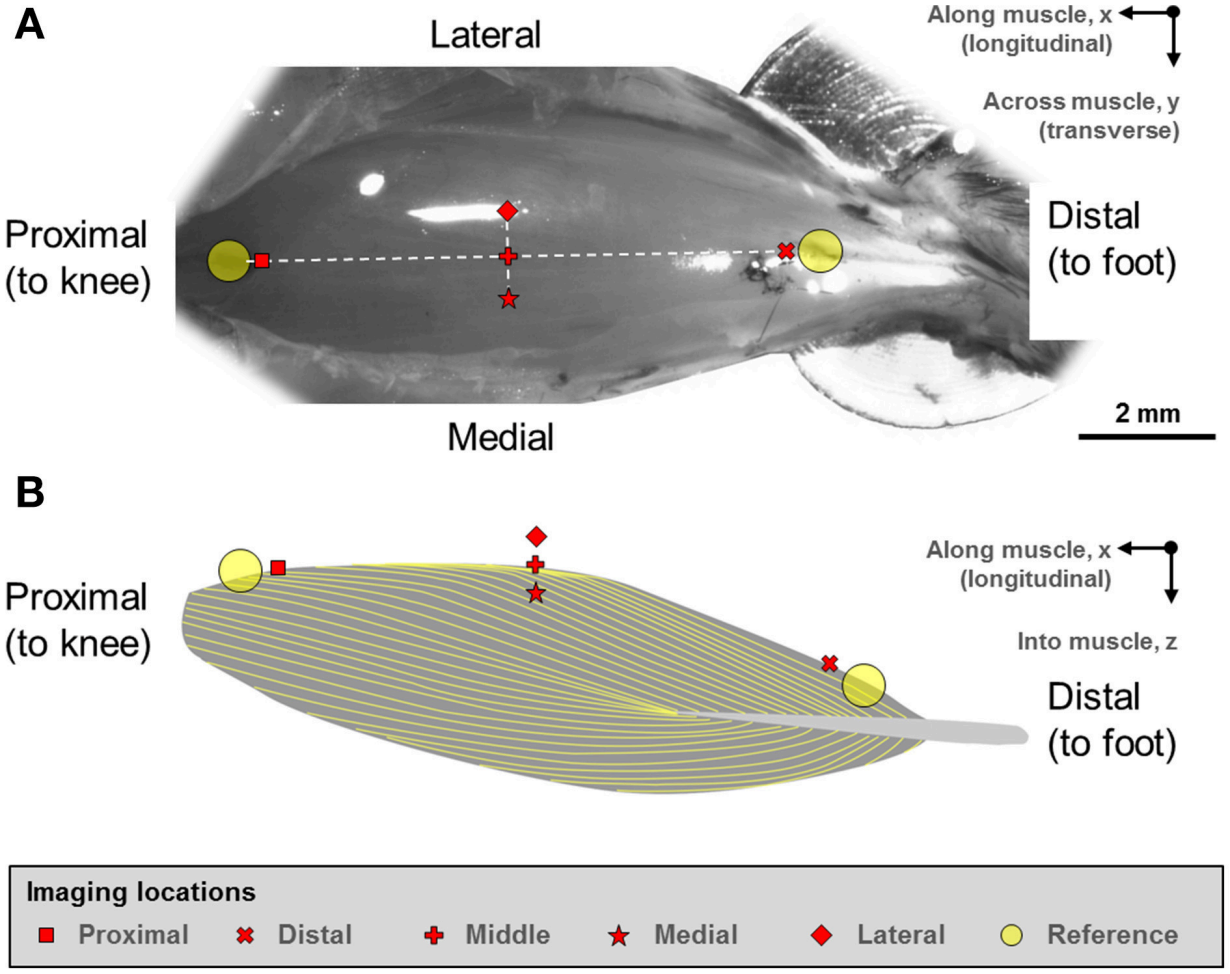
**(A)** Digital photograph of the mouse tibialis anterior (TA) muscle. **(B)** Schematic illustration of the muscle architecture in the mid-sagittal plane of the muscle (Heemskerk et al., 2005; Lovering et al., 2013). The fiber orientation is indicated by the yellow outlines. The sarcomere lengths of the mouse tibialis anterior muscle were imaged at five defined sites. Two reference points were first defined at the proximal and distal ends of the TA muscle. From these reference points, five imaging sites located along the longitudinal axis (“proximal,” “middle,” “distal” sites) and along the transverse axis (“medial,” “middle,” “lateral” sites) were identified on TA. The “middle” landmark was the mid-point of the straight line connecting the “proximal” and “distal” TA sites, while the “medial” and “lateral” landmarks were ~500 μm away from the “middle” landmark in the medial and lateral directions, respectively.

Two reference points were defined at the proximal and distal ends of the TA (Figure 1). From these reference points, five landmarks along the longitudinal axis of the muscle (“proximal,” “middle,” “distal” landmarks) and along the transverse axis of the muscle (“medial,” “middle,” “lateral” landmarks) were defined. The “middle” landmark was the mid-point of the straight line connecting the “proximal” and “distal” landmarks, while the “medial” and “lateral” landmarks were ~500 μm away from the “middle” landmark, respectively.

Second harmonic generation (SHG) imaging of the TA was performed using an upright, multi-photon excitation microscope (FVMPE-RS model, Olympus, Tokyo, Japan) equipped with a wavelength-tunable (680–1300 nm), ultrashort-pulsed (pulse width: < 120 fs; repetition rate: 80 MHz) laser (InSight DeepSee-OL, Spectra-Physics, CA, USA) and a 25 x/1.05 NA water immersion objective (XLPLN25XWMP2 model, Olympus, Tokyo, Japan). The TA was scanned using a laser wavelength of 800 nm. The resulting SHG signal emitted by the muscle was collected in the backward (epi-) direction using a band-pass filter at the harmonic frequency (FF01 400/40, Semrock Inc., NY, USA). The average power in the sample plane was varied from 14 to 40 mW in order to produce optimal images.

At each landmark, a series of planar images were acquired in the horizontal plane (xy-plane; imaging area: 159 × 159 μm; pixel size: 0.155 μm; bit-depth: 12; dwell time: 2 μs) along the objective axis (z-axis) that is perpendicular to the horizontal plane at 1 μm intervals. Image stacks were taken from the top 30 μm of the muscle (denoted as the surface zone) at all five imaging landmarks. For imaging landmarks that were located along the transverse axis of the muscle (“medial,” “middle,” and “lateral”), additional 30 μm thick image stacks were acquired from tissue that was 100 μm deep (denoted as the deep zone). The SHG imaging of the muscle was repeated at three ankle angles corresponding to full dorsiflexion (angle between foot and tibia ~50°), intermediate angle (~120°), and full plantarflexion (~180°). Only muscle images free of cardiac and respiratory motion artifacts were included in the image analysis.

### Image analysis

Due to the spindle-like shape of the mouse TA, image stacks were sequentially rotated in the xy- and xz-plane using “TransformJ: Rotate” plugin of ImageJ (National Institute of Health, MD, USA) in order to align the longitudinal axis of the muscle so that the epimysium was always parallel to the horizontal axis for ease of comparisons across the different landmarks (see Supplementary Materials, Figure S6).

**Figure 2:**
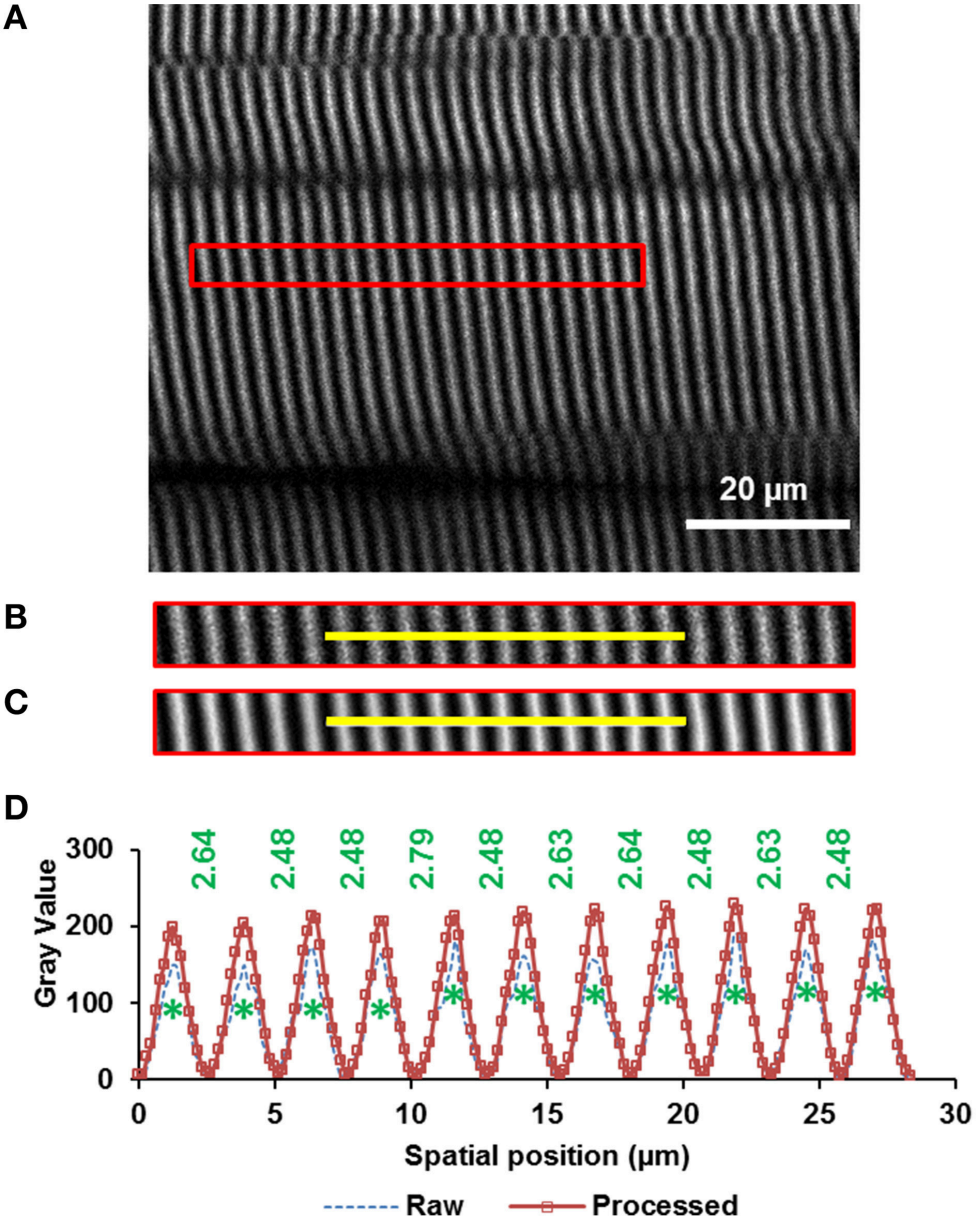
**Sarcomere lengths were measured from images obtained by second harmonic generation (SHG)**. The A-bands appear as white bands in the image. The image was taken from the middle site of the TA muscle of mouse #4 (see “Supplementary Materials,” Table S1) during full plantarflexion of the ankle joint. **(A)** A representative region of interest of 30 pixels wide that contains 20 sarcomeres (indicated by the red rectangle) was selected from the images (see text in Methods for details), **(B)** raw image, and **(C)** filtered image of the selected region. The image was filtered by a patch-based de-noising algorithm (Chatterjee and Milanfar, 2012), **(D)** Intensity profiles across the yellow line highlighted in the raw (dotted blue line) and filtered (rectangle symbols on solid red line) muscle image. The centroids of all A-bands (green asterisks) were identified using a custom-written MATLAB code. Individual sarcomere lengths were calculated as the distance (in micrometer) between adjacent A-band centroids.

From the rotated image stacks, four representative planar image bands of 30 pixels (~4.7 μm) wide that had good signal-to-noise ratio and contained 15–20 sarcomeres in series were selected from each of four sub-regions per site. By assuming a myofibril diameter of ~1.3 μm (Powers et al., 2016), we could determine that each planar image band contained 60–80 sarcomeres (~4 sarcomeres in parallel containing ~15–20 sarcomeres in series). Then, each image band was filtered by a patch-based de-noising algorithm (Chatterjee and Milanfar, 2012) using 50 patches of 5-pixel radius and an iteration number of 20. The resulting image band was further analyzed by a custom written MATLAB code that identified the centroids of the sarcomeric A-bands. Individual sarcomere lengths were measured as the distance between adjacent A-band centroids (Figure 2).

### Outcome measures

We quantified four main outcome measures: First, we determined the mean sarcomere lengths from the 240–320 sarcomere lengths measured at each location (distal, middle, proximal, medial, and lateral) in a muscle and this was done for surface lying sarcomeres at each site. For the deep lying sarcomeres, the mean sarcomere lengths were only measured at the medial, middle, and lateral sites. Second, we measured the mean sarcomere elongations associated with muscle lengthening from the fully dorsi-flexed to the fully plantar-flexed position at all muscle sites. Third, we determined the variations in sarcomere lengths at each site and expressed them as local standard deviations (*SD*) and local coefficients of variation (*CV* = standard deviation/mean); and fourth, we determined the variations in sarcomere lengths across the entire TA and expressed them as global *SD* and global *CV*. These last variations in sarcomere lengths were determined along the TA (using the proximal, middle, and distal site values) and across the TA (using the medial, middle and lateral sites).

### Statistical analysis

Descriptive statistical analysis was performed on the data of all muscles (*n* = 5), by calculating means ± 1 standard deviations (*SD*) of sarcomere lengths at each muscle site (local analysis) and averaged across all sites of the TA (global analysis). Means of mean sarcomere length, sarcomere elongation, local *SD*, local *CV*, global *SD*, and global *CV*, were compared between the different sites on the muscle and for the three different muscle lengths using two-way repeated measures ANOVA (with Bonferroni adjustment). In addition, means of mean sarcomere lengths were also compared between the surface zone sarcomeres and the deep zone sarcomeres and for the three muscle lengths using two-way repeated measures ANOVA (with Bonferroni adjustment) with α = 0.05 (SPSS 21, SPSS Inc., IL, USA).

## Results

The average mass of the mice was 26.3 ± 2.3 g. Surface zone and deep zone sarcomeres had similar average lengths (data not shown). Unless specified otherwise, the results presented in the following paragraphs are from the surface zone sarcomeres exclusively.

**Figure 3:**
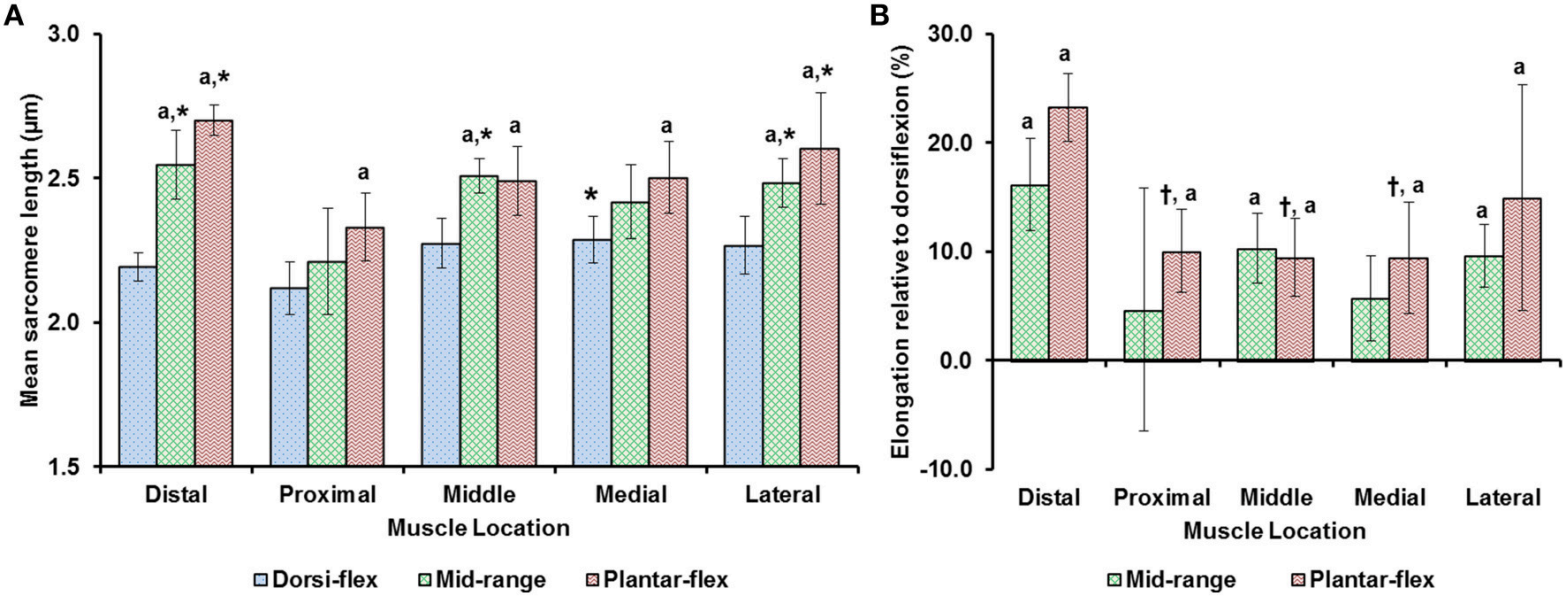
**(A)** Mean lengths of surface zone sarcomeres at the five target sites (“distal,” “proximal,” “middle,” “medial,” and “lateral”) of the tibialis anterior (TA) muscle at full dorsiflexion, intermediate ankle angle, and full plantarflexion. **(B)** Elongation of sarcomeres relative to the sarcomere lengths at full dorsiflexion. The graphs show the average values of the mean sarcomere lengths **(A)** and elongations **(B)** for the five mice. Sarcomere lengths varied with locations on the TA. When the TA was lengthened, sarcomeres at all sites elongated but in a location-dependent manner. Sarcomeres at the “proximal” TA site were shorter compared to sarcomeres at the other sites. When the TA was elongated, sarcomeres at the “distal” TA site lengthened more than sarcomeres at the other sites. ^*^Indicates significant differences in sarcomere length compared to the sarcomeres at the proximal TA site for a particular ankle position (*p* < 0.05). ^a^Shows significant differences in sarcomere length or sarcomere elongation compared to the corresponding length at full dorsiflexion (*p* < 0.01). ^†^Represents significant differences in sarcomere elongation compared to the sarcomeres at the distal TA site (*p* < 0.05; see section Statistical Analysis in the “Methods” for more details).

Average sarcomere lengths varied substantially for the different TA sites. At full dorsiflexion (shortest muscle length), mean sarcomere lengths ranged from 2.1 to 2.3 μm, with sarcomeres at the “medial” site being significantly longer than sarcomeres at the “proximal” site (Figure 3). When the TA was stretched passively by moving the ankle from full dorsiflexion to full plantarflexion, sarcomeres at the different TA sites were elongated between 10 and 25% of their original length. Going from the fully dorsi-flexed to the intermediate ankle position, only the sarcomere lengths at the “distal,” “middle,” and “lateral” sites were elongated significantly while those at the proximal and medial sites elongated to a smaller and non-significant degree (Figures 3A,B).

**Figure 4:**
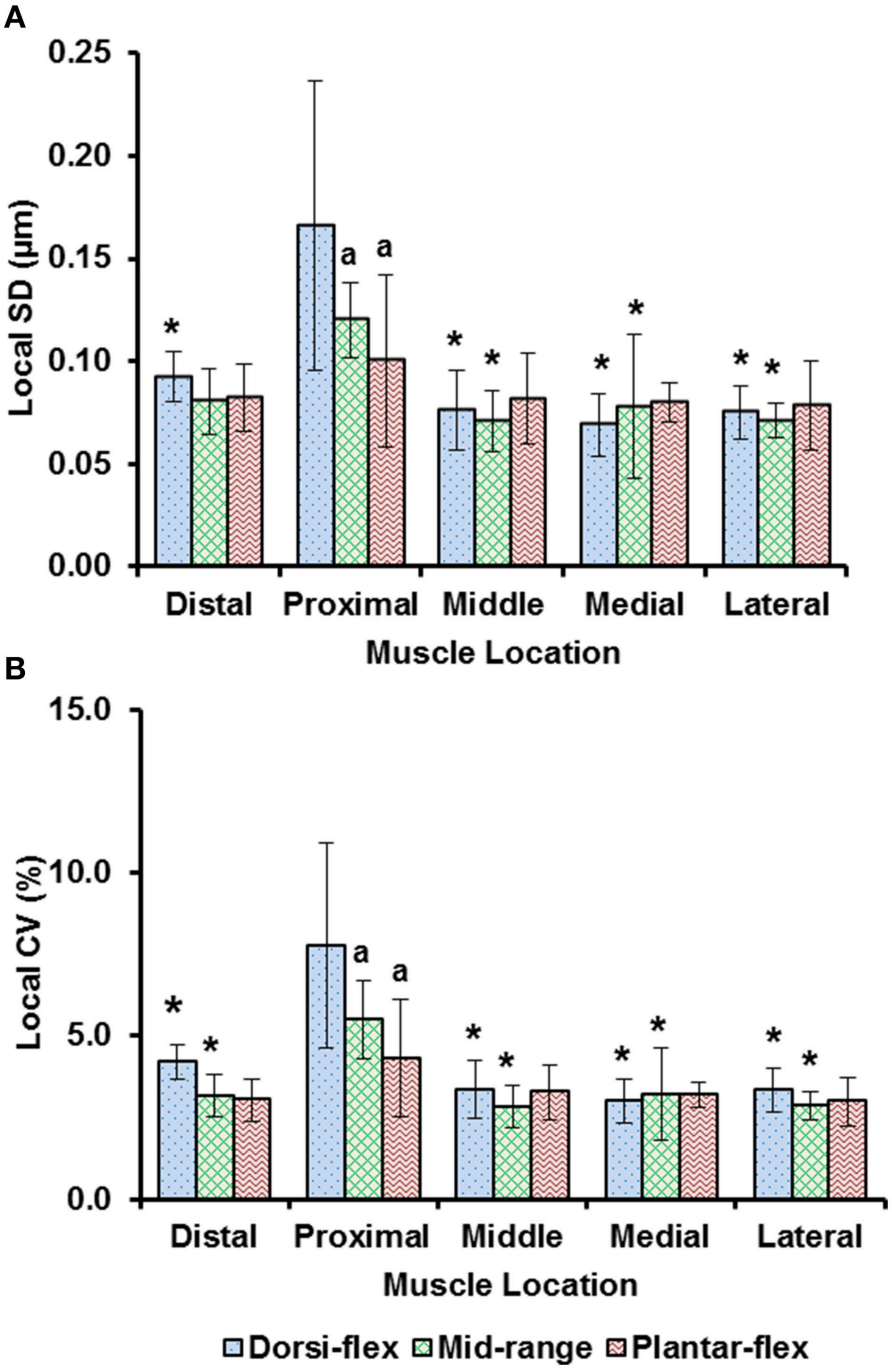
**Local dispersion of sarcomere lengths at full dorsiflexion, intermediate ankle angle, and full plantarflexion given by (A) the local standard deviation (***SD***) and, (B) the local coefficient of variation (***CV***)**. For each TA site of 159 × 159 μm, the local dispersion of sarcomere lengths was measured from four representative image bands that were of 4.7 μm wide and contained 15–20 sarcomeres in series. The graphs show the average values of the local *SD*s (a) and local *CV*s (b) for the five mice. Sarcomeres at the “proximal” TA site had higher local *SD* and local *CV* compared to sarcomeres at the remaining TA sites. ^*^Indicates significant differences in local *SD* and local *CV* compared to the sarcomeres at the proximal TA site and a given ankle joint angle (*p* < 0.05). ^*a*^Indicates significant differences in local *SD* and local *CV* compared to sarcomeres in the fully dorsi-flexed position (*p* < 0.05; see Section Statistical analysis in the “Methods” for more details).

**Figure 5:**
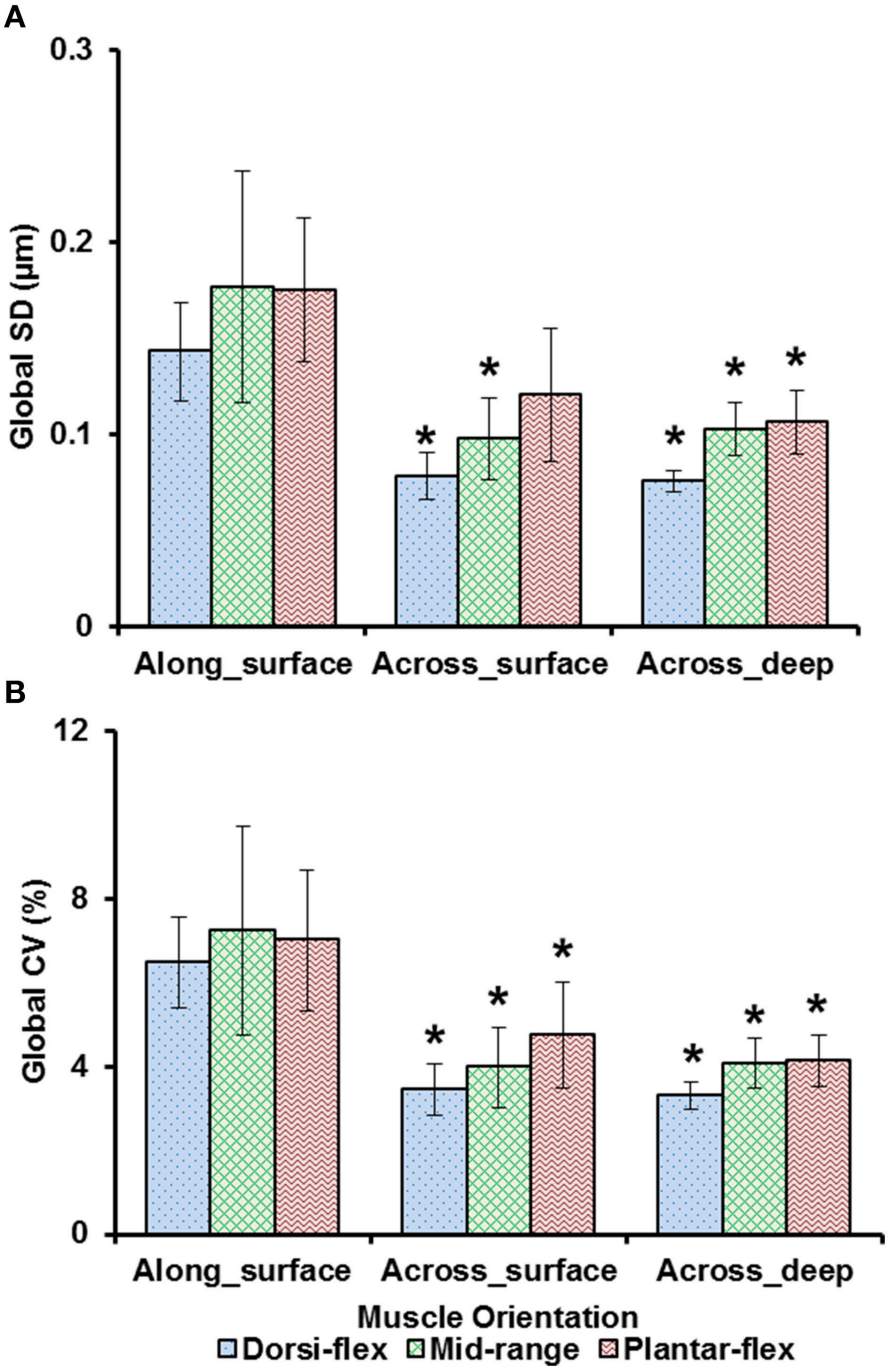
**Global dispersion in sarcomere lengths for surface zone and deep zone sarcomeres at full dorsiflexion, intermediate ankle angle, and full plantarflexion. (A)** Global standard deviation (*SD*) and, **(B)** global coefficient of variation (*CV*). Sarcomeres from the surface zone were pooled based on TA site, either into measurements along the longitudinal axis (“proximal,” “middle,” and “distal” TA sites) or along the transverse axis of the muscle (“medial,” “middle,” and “lateral”). In addition, sarcomeres from the deep zone that were located along the transverse axis of the TA were analyzed for their dispersions. The graphs show the average of the global *SD*s and global *CV*s from the five mice tested. Surface zone sarcomeres along the longitudinal axis of the TA had greater dispersions of length compared to sarcomeres that were aligned along the transverse axis of the TA. ^*^Indicates significant differences in global *SD* and global *CV* compared to surface zone sarcomeres that were located along the longitudinal axis of the TA at a given ankle angle (*p* < 0.05; see Section Statistical Analysis in the “Methods” for more details).

Sarcomere lengths varied along fibers at each TA site and also varied between the five TA sites. Locally, sarcomere length in series had standard deviations (*SD*) of ~0.1 μm, and the local coefficient of variation (*CV*) was ~5% at all TA sites, except for the “proximal” site where sarcomeres had higher local *SD* (~0.18 μm) and local *CV* of (~8%) at full dorsiflexion. These variations in sarcomere lengths at the “proximal” TA site decreased as the TA was stretched from the dorsi-flexed, to the intermediate, and the full plantar-flexed ankle position (Figures 4A,B). Sarcomere length variations across the different TA sites were greater along the muscle than across the muscle with global *SD* of ~0.20 μm (along TA) vs. ~0.12 μm (across TA) and global *CV* of ~8% (along TA) vs. ~5% (across TA; Figure 5).

**Figure 6:**
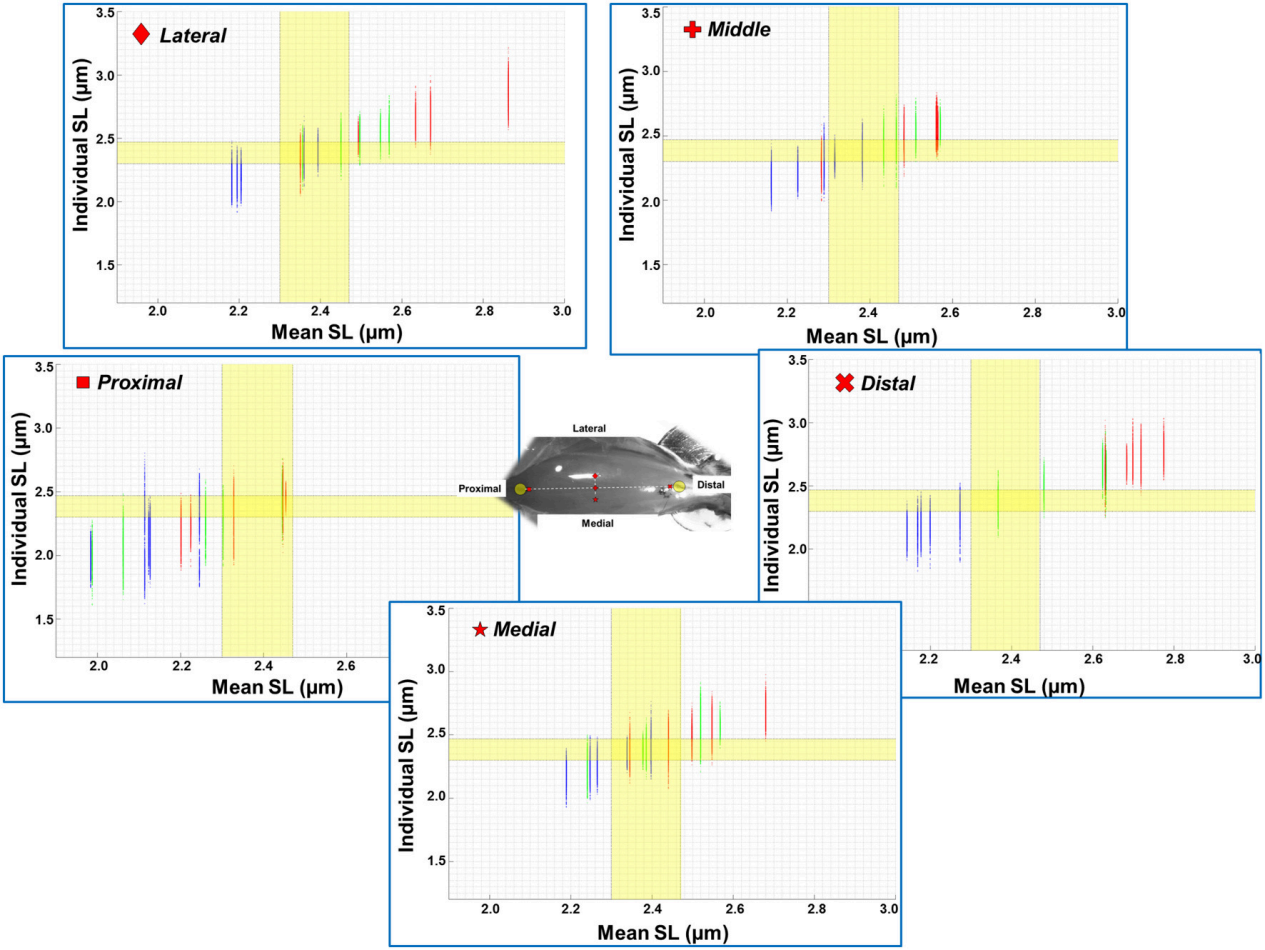
**Scatter plot of individual sarcomere lengths against mean sarcomere lengths across all five TA sites where measurements were made (***n*** = 5)**. Results are shown for the ankle in a position of full dorsiflexion (blue), intermediate angle (green), and full plantarflexion (red). The optimal range of sarcomere lengths of mouse TA was calculated from the known lengths of the thin and thick myofilaments (Gokhin et al., 2014). The optimal length is identified by the yellow bands (2.30–2.47 μm). The lengths of serially arranged sarcomeres varied substantially even in the small local regions. Individual sarcomere length variability may be underestimated when sarcomere lengths are expressed as mean values across a region, as evidenced by the wider range of sarcomere lengths along the y-axis (individual lengths) compared to those along the x-axis (mean lengths). All data points from all five animals (corresponds to 1200–1500 sarcomeres) are shown in each of the graphs. See Supplementary Materials for scatter plots of each imaging site.

**Figure 7:**
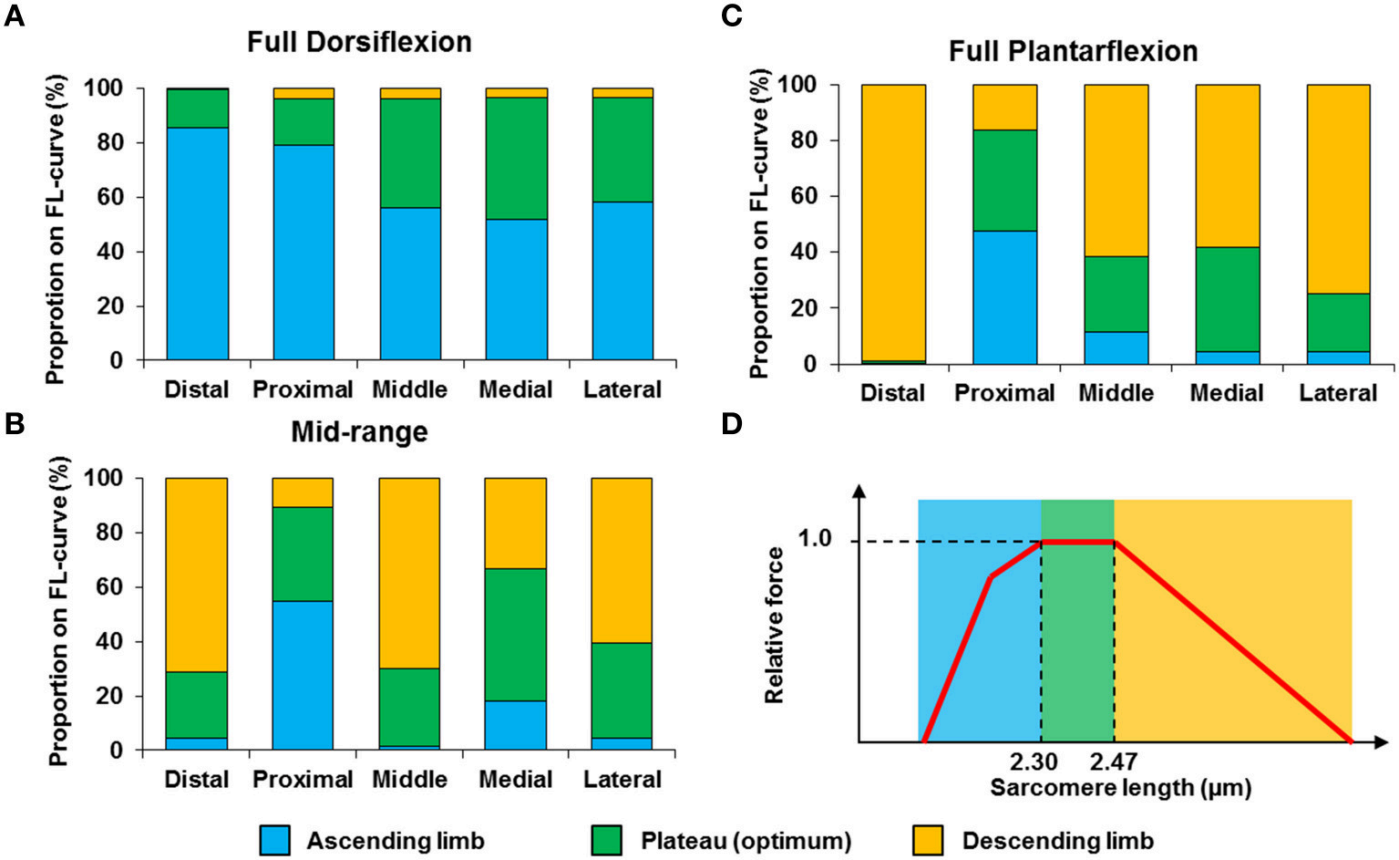
**Proportion of sarcomeres with lengths that fall onto the ascending limb (blue bars), plateau region (green bars) and descending limb (orange bars) of the theoretical force-length curve at (A) full dorsiflexion, (B) intermediate ankle angle, and (C) full plantarflexion**. A representative sarcomere force-length curve (red curve) of mouse TA is shown in **(D)**. The theoretical optimal sarcomere length ranges from 2.30 to 2.47 μm (Walker and Schrodt, 1974; Gokhin et al., 2014).

The mean, standard deviation, minimum value, and maximum value of sarcomere length for all five TA sites and all five muscles are summarized in Table S1 (See Supplementary Material). In a region of 159 × 159 μm^2^, the local difference between the longest and the shortest sarcomere were as high as 1 μm (Table S1). The great range of sarcomere lengths is illustrated in the scatter plot of individual sarcomere lengths against the mean sarcomere lengths pooled across all muscles and all TA sites analyzed (Figure 6). Scatter plots of individual sarcomere length against mean sarcomere length of the individual TA sites are also shown, for completeness (Figures S1–S5; Supplementary Material). From the pooled data, the proportion of sarcomeres on the ascending limb, plateau region, and descending limb of the theoretical force-length curve were also quantified (Figure 7).

## Discussion

In the current study, the distribution of passive muscle sarcomere lengths over the full range of motion of intact, living whole muscle was systematically investigated using non-linear SHG imaging. With multi-photon microscopy, muscle striation patterns (I- and A-bands) can be visualized through the interaction of the high intensity laser beam with the myosin thick filaments that are organized non-centrosymmetrically (Plotnikov et al., 2006). Since a strong SHG signal can be detected easily even at a low laser power (Llewellyn et al., 2008; Cromie et al., 2013), individual sarcomere lengths can be measured directly from the whole muscle *in vivo* in a manner that does not require contact with the muscle that could affect local sarcomere lengths and sarcomere lengths non-uniformities. Mice were kept alive throughout the experiment to avoid muscle rigor (Burkholder and Lieber, 2001). The mouse TA was chosen for three reasons: (1) it provides easy access for imaging, (2) although TA muscle is a pennate muscle (Heemskerk et al., 2005; Lovering et al., 2013), its muscle fibers run mostly along the muscle surface, which facilitates imaging of sarcomeres at any location; and (3) TA is a single-joint muscle, so its muscle tendon unit length is uniquely given by the ankle joint angle. As imaging of each planar image took ~2.1 s, any cardiac or respiratory movements during muscle scanning may cause a distortion of the planar image. We controlled for possible motion artifacts by deep anesthesia of the mice to slow the breathing and heart rates, by fixing the knee rigidly to a custom-made knee clamp that isolate the lower leg and TA muscle from cardiac and respiratory movements, and by carefully selecting images free of motion artifacts during image processing.

We found that sarcomere lengths varied substantially between TA sites (Figure 3). In general, sarcomeres at the “proximal” TA site were shorter than sarcomeres at the other sites (Figure 3A). When the TA was passively stretched, the amount of sarcomere lengthening was location-dependent, with sarcomeres at the “distal” TA site being stretched more (by up to 25%) than sarcomeres at the other sites (Figure 3B). The location-dependent sarcomere elongations can be attributed to the muscle architecture of TA, with the muscle being unipennate on the proximal end but bipennate on the distal end (Heemskerk et al., 2005). Although, the muscle length changes due to changes in ankle angle were not measured, previous studies suggest that TA muscle elongations are linearly related to changes in ankle angles (Burkholder and Lieber, 1998, Figure 4). However, sarcomere length elongations were not linear, with increases in sarcomere lengths being much greater when going from the dorsi-flexed to the mid ankle position compared to when going from the mid ankle to the fully plantar-flexed position (Figure 3B). Such non-linear elongation of sarcomeres along TA muscle may be caused by the spindle-like shape of the muscle (Figure 1B). The distal muscle has a smaller cross sectional area compared to the middle and proximal muscle. Therefore, a passive stretch of the muscle may result in greater sarcomere elongations in the distal compared to the middle and proximal region as there are fewer sarcomeres in parallel, and therefore, the muscle may be less stiff in the distal compared to the middle and proximal regions. At the “middle” TA site, sarcomeres were not stretched, on average, when the ankle was moved from the intermediate angle to the fully plantar-flexed ankle angle.

Previous studies typically investigated sarcomere length locally from a small region of the muscle (Cutts, 1988; Llewellyn et al., 2008; Cromie et al., 2013). In the current study, sarcomere lengths in mouse TA muscle were measured locally from a small region (159 × 159 μm^2^) of the muscle, and globally from distinct sites along and across the entire muscle in order to quantify the variability of sarcomere lengths. Locally, we found that the standard deviation (*SD*) and coefficient of variation (*CV*) of sarcomere lengths were ~0.1 μm and 5%, respectively (Figure 4). These values are comparable to previous *in vivo* studies using whole muscle (Llewellyn et al., 2008; Cromie et al., 2013), *ex vivo* studies using muscle biopsies (Plotnikov et al., 2008), *in vitro* studies using muscle fibers (Infantolino et al., 2010) and *in vitro* studies using isolated myofibrils (Rassier and Pavlov, 2010; Johnston et al., 2016). It should be noted that even though the *SD* and *CV*-values are relatively small, the differences between the longest and the shortest sarcomeres from these small local regions can be as high as 1 μm, which corresponds to about 40% of the average sarcomere length (Table S1, Figure 6). When the data were pooled to include the entire muscle, the global dispersion of sarcomere lengths, particularly along the longitudinal axis of the muscle, was higher than the local sarcomere length non-uniformities. The global *SD* and *CV* for surface zone sarcomeres located along the longitudinal axis of the TA were ~0.2 μm and ~8%, respectively (Figure 5).

We also looked into the length range over which sarcomeres operate at rest. Based on the classic cross-bridge theory, the optimal operating length of sarcomeres of the mouse TA was calculated to range between 2.30 and 2.47 μm (actin filament: 1.1 μm; (Gokhin et al., 2014), myosin filament: 1.6 μm, Z-disks: 0.1 μm, bare zone: 0.17 μm). Although sarcomeres are thought to produce the highest force and power at their optimal lengths (Gordon et al., 1966), sarcomeres did not operate exclusively at their optimal lengths (Figures 6, 7). At full dorsiflexion, sarcomeres of the mouse TA were found to be mostly on the ascending limb and plateau region of the force-length curve. With increasing TA lengths, the sarcomeres were shifted toward the plateau region and the descending limb of the force-length curve. However, under no conditions were the sarcomeres observed to stay exclusively at their optimal lengths with the changes in TA length.

The results of this study indicate that neither local nor global sarcomere lengths are uniform in the passive mouse TA muscle. The local non-uniformities in sarcomere lengths are similar to those observed in single fibers (Huxley and Peachey, 1961; Infantolino et al., 2010), isolated myofibrils (Rassier and Pavlov, 2010; Johnston et al., 2016), and locally, also in entire muscles (Llewellyn et al., 2008; Cromie et al., 2013). This result, combined with the findings from other studies, suggests that sarcomere lengths are naturally non-uniform in muscles, and that the results observed here are likely applicable across vertebrate skeletal muscles. The range of sarcomere lengths observed locally differed by more than 1.0 μm, which is more than 40% of the optimal sarcomere length.

Novel to the literature is the fact that the mean sarcomere lengths measured at the different sites of the mouse TA also differ, and that sarcomere elongations with muscle lengthening are non-uniformly distributed across the muscle. Sarcomere length changes of as little as 10% and as much as 25% were observed when the TA was moved through its natural *in vivo* length range. The functional implications of these local and global sarcomere length non-uniformities remain unknown as it has not been possible to measure sarcomere length in active muscle simultaneously at different muscle sites. Furthermore, sarcomere lengths and associated length non-uniformities have not been explored in the context of active, dynamic properties, such as the force-length or force-velocity properties. These questions need to be addressed in future works. However, it is safe to assume that based on the findings of this study, it seems overly simplistic to measure sarcomere lengths of a muscle at a single location (Cutts, 1988; Takahashi et al., 2007), and a single muscle lengths and infer sarcomere lengths for other locations on the muscle or for other muscle lengths.

There are limitations in this study that need careful consideration when interpreting our results. First, several layers of skin and fascia covering the TA were removed for optimal SHG imaging. However, this process did not result in visible changes to the structure of the muscle (Figure 1), thus we assume that sarcomere length measurements obtained in this manner are identical to those that would be obtained in the fully intact muscle. Second, due to the limitations in tissue penetration of the SHG approach, the deep zone sarcomere length could only be obtained from a tissue depth of up to 130 μm.

Despite these limitations, we provide novel insight into the distribution of sarcomere lengths over an entire muscle. Future studies should quantify sarcomere length variability in intact whole muscles that are activated. *In vitro* studies with isolated myofibrils suggest that sarcomere length non-uniformity increases from the passive to the active state (Telley et al., 2006; Rassier and Pavlov, 2010; Johnston et al., 2016). However, this finding might not hold for entire muscle preparations. Also, although TA is essentially a fast-twitch muscle with virtually no type I fibers (Burkholder et al., 1994; Allen et al., 2001), type II fibers can be divided into type IIa, type IIx, and type IIb fibers. Future measurements should investigate the influence of muscle fiber type on the sarcomere length variation. Finally, future evaluation of individual sarcomere lengths should be performed for dynamic contractions of everyday movements while following a small subset of sarcomeres during these contractions. At present, such data remain elusive and much technical development will be required before we can follow a set of sarcomeres during normal every day contractions in an intact muscle continuously.

In summary, to our knowledge, this is the first study in which passive sarcomere length variability was determined across an entire muscle using a non-contact approach. We showed that sarcomere lengths varied substantially within small regions of the muscle and also for different sites across the muscle. Furthermore, sarcomere elongations were non-linear with muscle length and they were highly dependent on the precise location of the sarcomeres on the muscle: the highest sarcomere stretches occurring near the distal myotendinous junction. As a result, muscle mechanics derived from sarcomere length measured from a small region of a muscle may not represent well the sarcomere length and associated functional properties of the entire muscle.

## Author contributions

Substantial contributions to the conception or design of the work; or the acquisition, analysis, or interpretation of data for the work: EM, RF, SS, ZA, WH. Drafting the work or revising it critically for important intellectual content: EM, RF, SS, ZA, WH. Final approval of the version to be published: EM, RF, SS, ZA, WH. Agreement to be accountable for all aspects of the work in ensuring that questions related to the accuracy or integrity of any part of the work are appropriately investigated and resolved: EM, RF, SS, ZA, WH.

## Funding

This study was supported by the Alberta AI-HS Team grant on osteoarthritis (grant number: 200700596), AI-HS postdoctoral fellowship (grant number: 10013510), NSERC CREATE training program of Biomedical Engineers for the Twenty-first Century (grant number: CREAT/371280-2009), the Canadian Institutes of Health Research (CIHR; grant number: MOP-111205 and 140824), the Canada Research Chair Programme, and the Killam Foundation.

### Conflict of interest statement

The authors declare that the research was conducted in the absence of any commercial or financial relationships that could be construed as a potential conflict of interest.
