# Supplementary material for: In vivo Sarcomere Lengths and Sarcomere Elongations Are Not Uniform across an Intact Muscle
Source: Front Physiol. 2016 May 25;7:187. doi: 10.3389/fphys.2016.00187 (PMC4879144; doi:10.3389/fphys.2016.00187)
Supplement: Supplementary file 1 [file Figure1.docx]

Supplementary Material

In vivo sarcomere lengths and sarcomere elongations are not uniform across an intact muscle

Eng Kuan **Moo**, Rafael **Fortuna**, Scott **Sibole**, Ziad **Abusara**, *Walter **Herzog**

*** Correspondence:** Walter Herzog: wherzog@ucalgary.ca

# Supplementary Figures


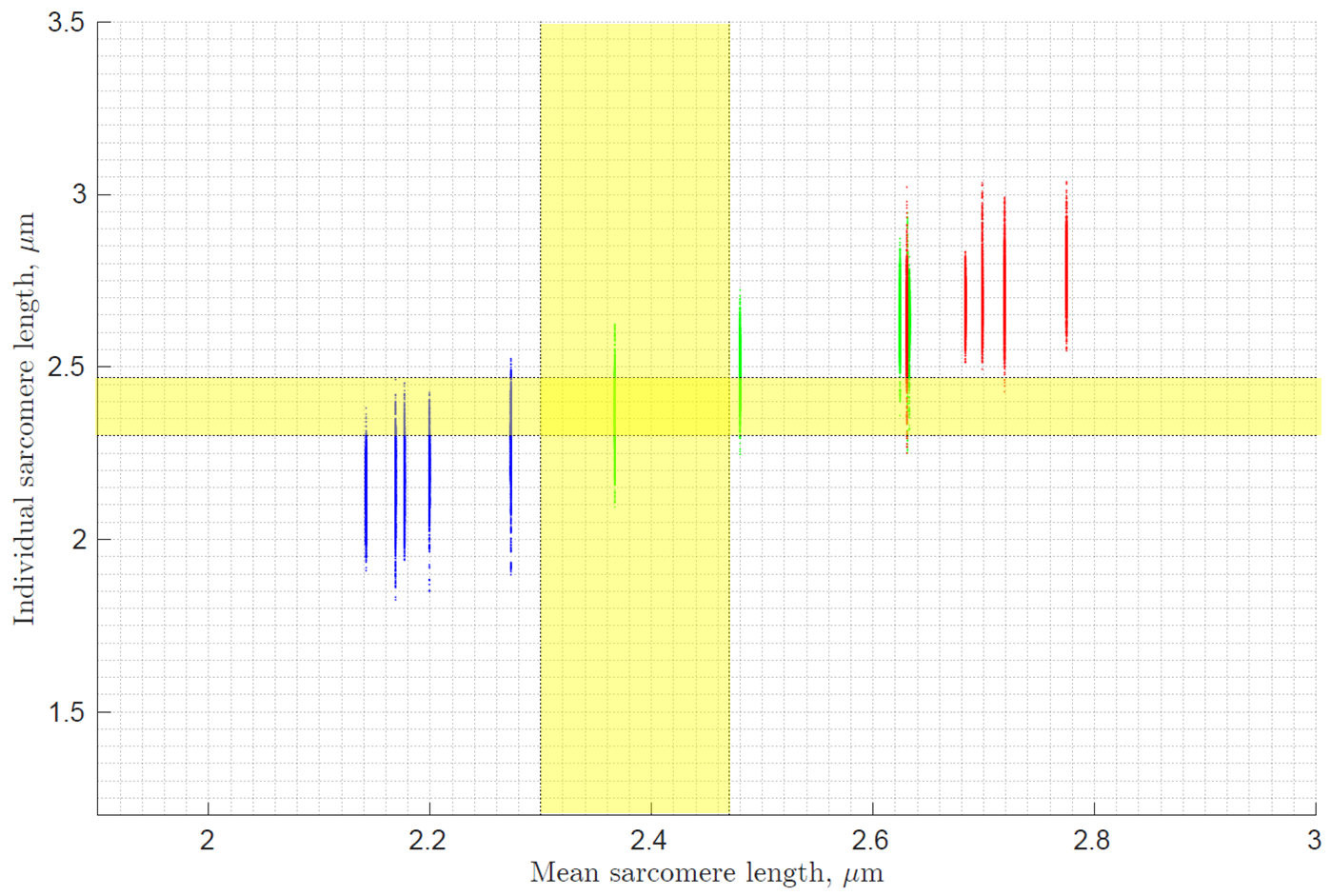


Figure S1: Scatter plot of individual sarcomere lengths against mean sarcomere lengths for the ‘distal’ TA sites (n = 5) at full dorsiflexion, intermediate ankle angle and full plantarflexion. The theoretical optimal sarcomere lengths are highlighted in yellow. When moved passively from shortest to longest muscle lengths, the mean sarcomere lengths (x-axis) varied from 2.14 – 2.77 µm while individual sarcomere lengths (y-axis) varied from 1.82 – 3.04 µm. Mean sarcomere lengths only increased distinctly for the distal muscle location, but not for the other locations measured in this study.


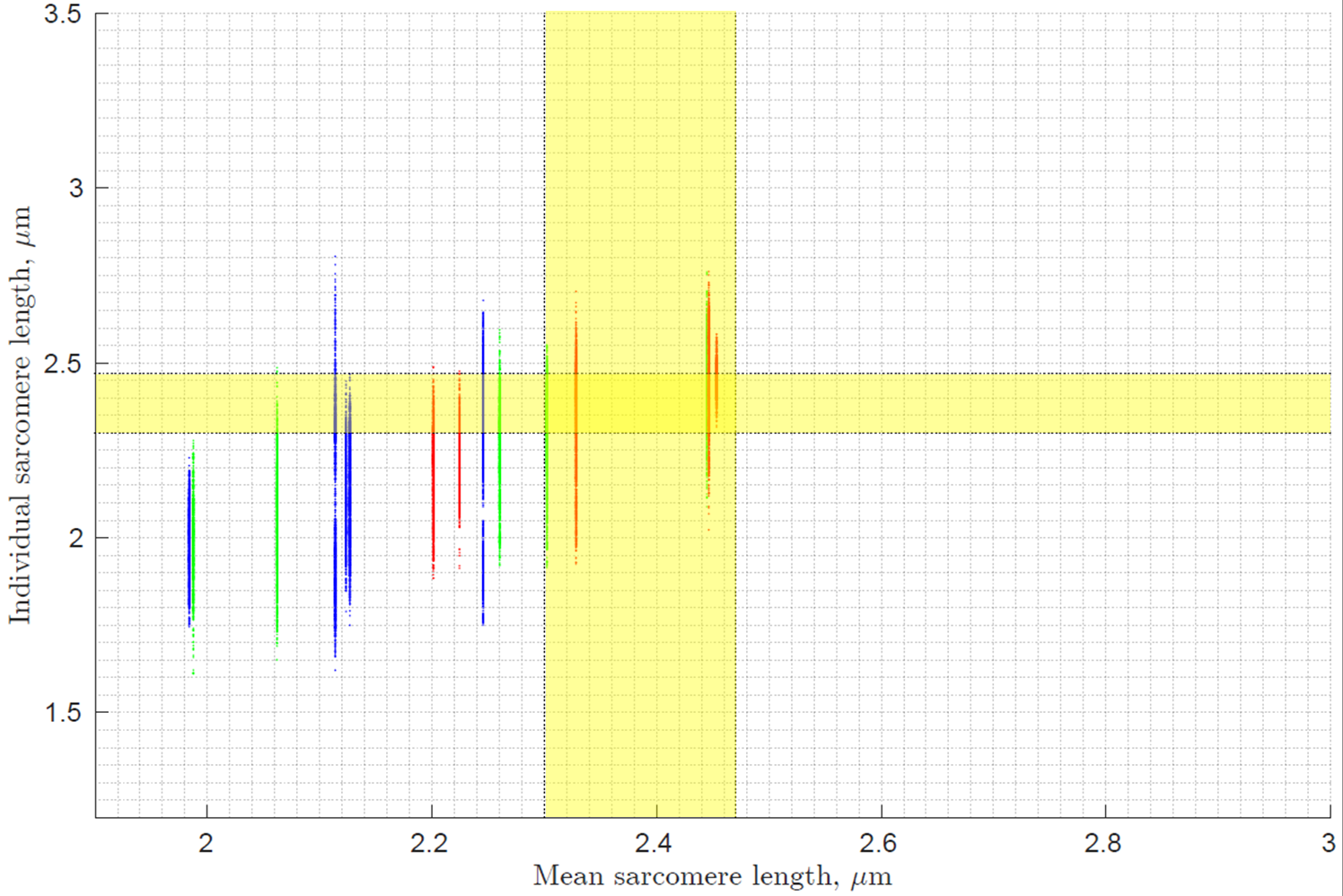


Figure S2: Scatter plot of individual sarcomere lengths against mean sarcomere lengths for the ‘proximal’ TA site (n = 5) at full dorsiflexion, intermediate ankle angle and full plantarflexion. The theoretical optimal sarcomere lengths are highlighted in yellow. When moved passively from shortest to longest muscle lengths, the mean sarcomere lengths (x-axis) varied from 1.98 – 2.45 µm while individual sarcomere lengths (y-axis) varied from 1.61 – 2.80 µm.


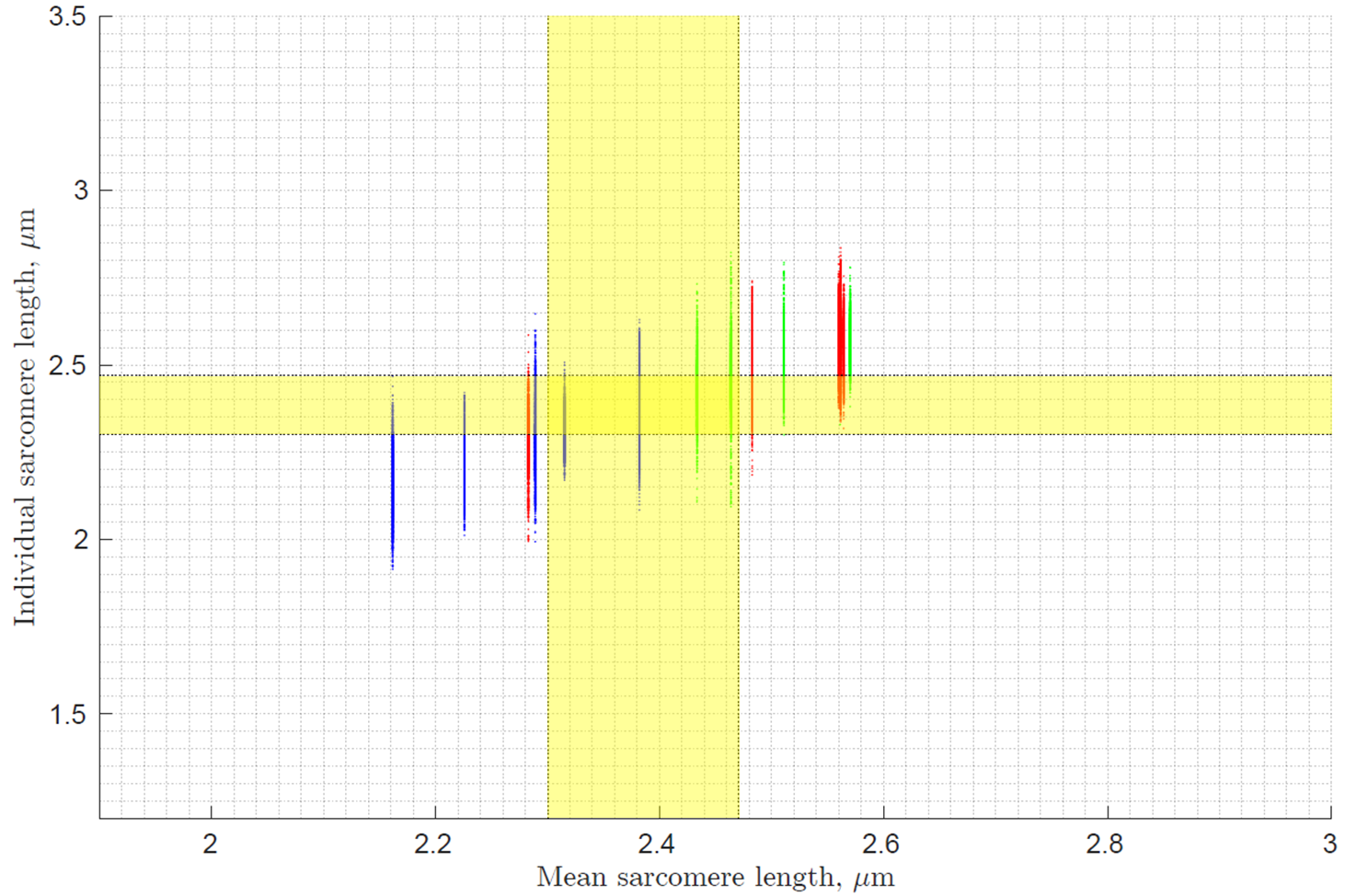


Figure S3: Scatter plot of individual sarcomere lengths against mean sarcomere lengths for the ‘middle’ TA site (n = 5) at full dorsiflexion, intermediate ankle angle and full plantarflexion. The theoretical optimal sarcomere lengths are highlighted in yellow. When moved passively from shortest to longest muscle lengths, the mean sarcomere lengths (x-axis) varied from 2.16 – 2.57 µm while individual sarcomere lengths (y-axis) varied from 1.91 – 2.84 µm.


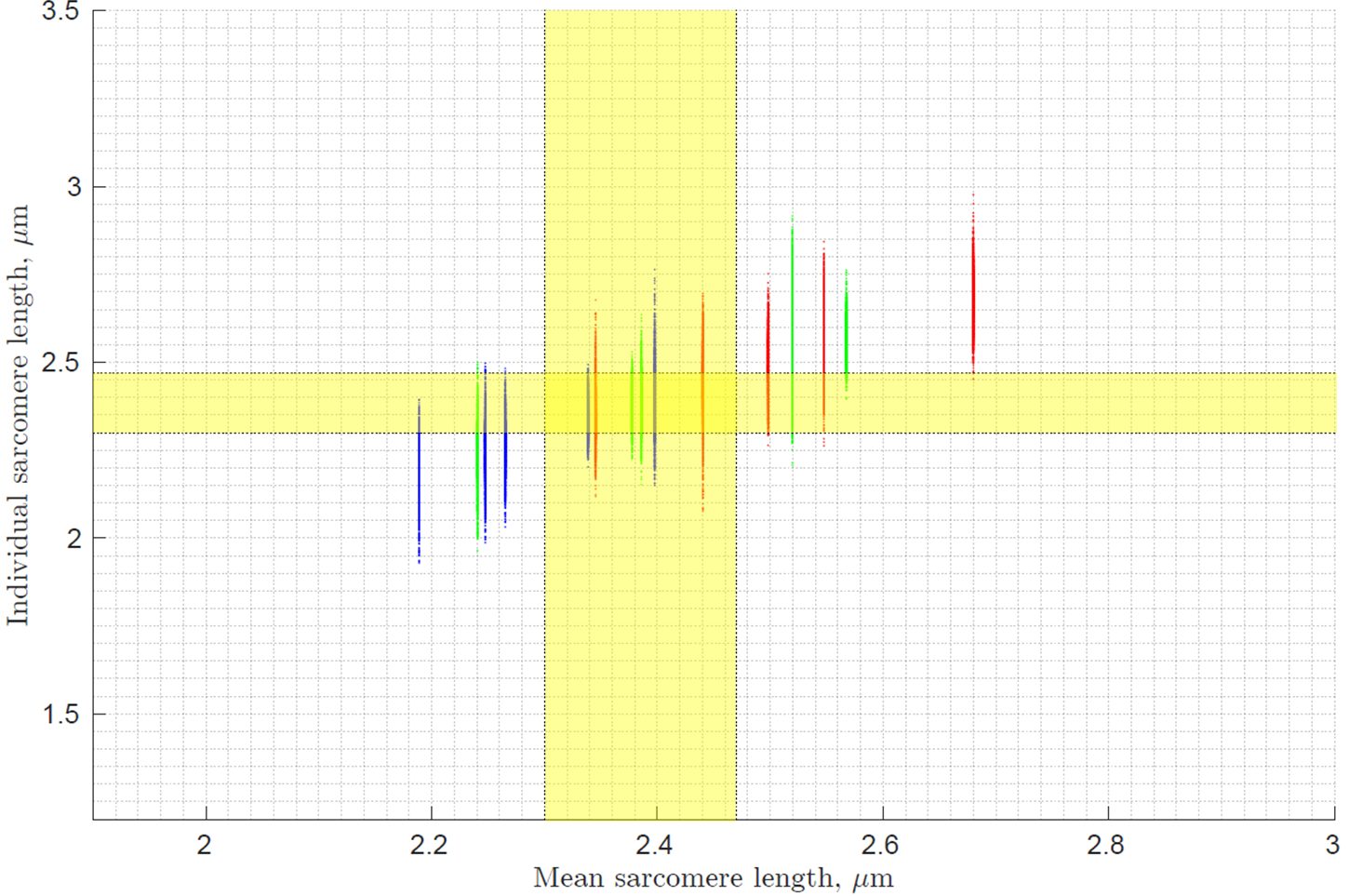


Figure S4: Scatter plot of individual sarcomere lengths against mean sarcomere lengths for the ‘medial’ TA site (n = 5) at full dorsiflexion, intermediate ankle angle and full plantarflexion. The theoretical optimal sarcomere lengths are highlighted in yellow. When moved passively from shortest to longest muscle lengths, the mean sarcomere lengths (x-axis) varied from 2.19 – 2.68 µm while individual sarcomere lengths (y-axis) varied from 1.93 – 2.98 µm.


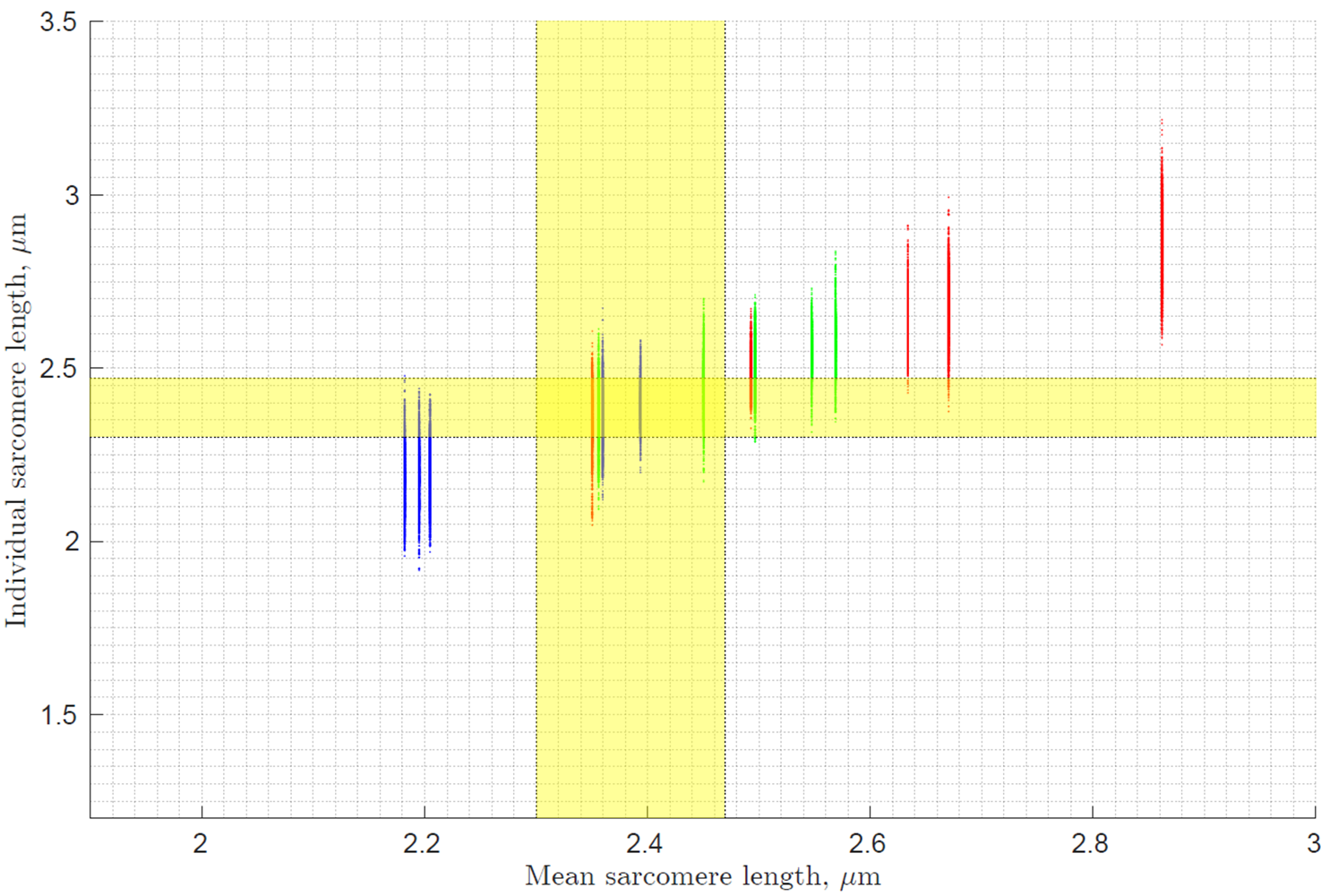


Figure S5: Scatter plot of individual sarcomere lengths against mean sarcomere lengths for the ‘lateral’ TA site (n = 5) at full dorsiflexion, intermediate ankle angle and full plantarflexion. The theoretical optimal sarcomere lengths are highlighted in yellow. When moved passively from shortest to longest muscle lengths, the mean sarcomere lengths (x-axis) varied from 2.18 – 2.86 µm while individual sarcomere lengths (y-axis) varied from 1.92 – 3.22 µm.


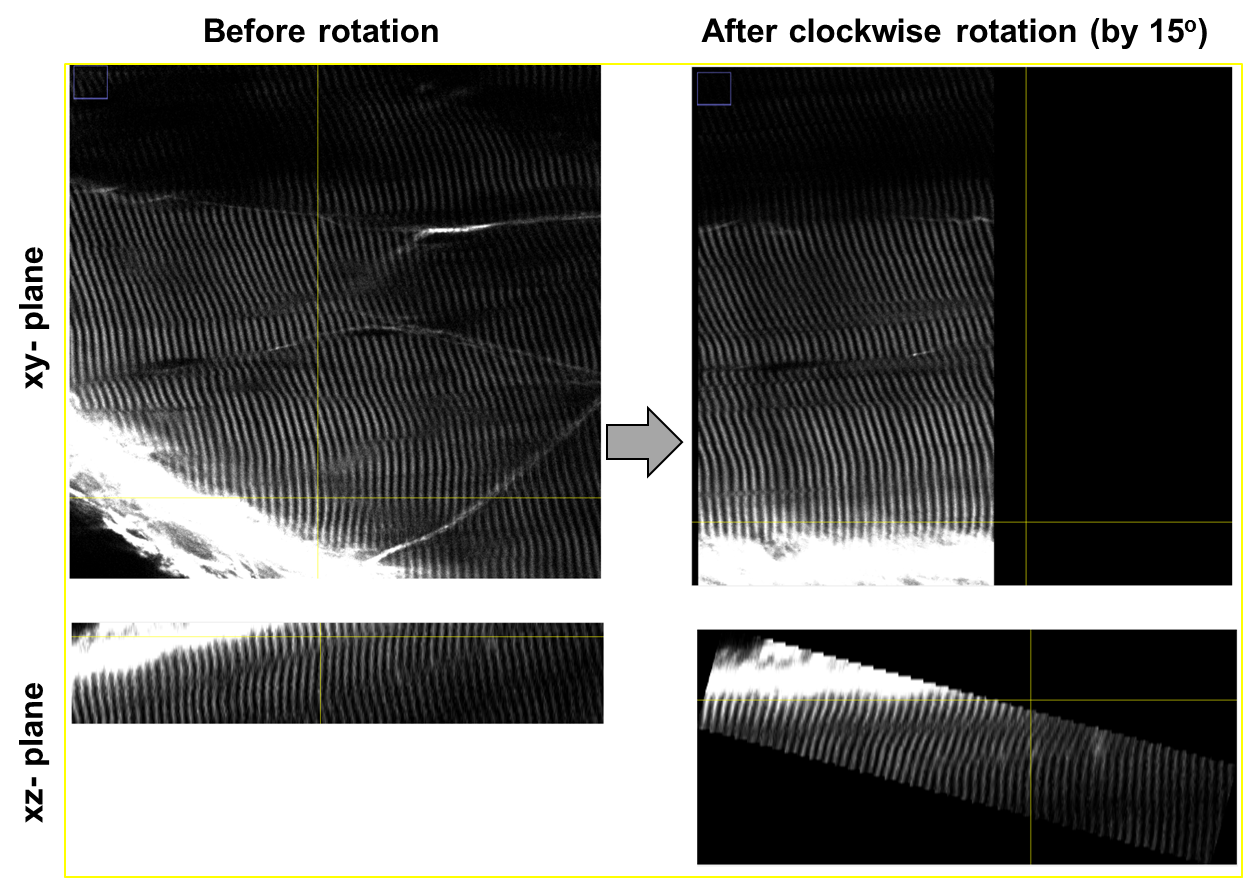


Fig. S6 Orthogonal view of the muscle image stack showing the views from the xy-plane (horizontal plane) and from the xz-plane (vertical plane). The digital rotation was applied to the image stack so that the epimysium (indicated by the bright bands) was parallel to the horizontal plane in the xz-plane.
