# Supplementary material for: In vivo Sarcomere Lengths and Sarcomere Elongations Are Not Uniform across an Intact Muscle
Source: Front Physiol. 2016 May 25;7:187. doi: 10.3389/fphys.2016.00187 (PMC4879144; doi:10.3389/fphys.2016.00187)
Supplement: Supplementary file 2 [file Table1.DOCX]

Supplementary Material

In vivo sarcomere lengths and sarcomere elongations are not uniform across an intact muscle

Eng Kuan **Moo**, Rafael **Fortuna**, Scott **Sibole**, Ziad **Abusara**, *Walter **Herzog**

*** Correspondence:** Walter Herzog: wherzog@ucalgary.ca

# Supplementary Table

Table S1: Mean, standard deviation, minimum value and maximum value of sarcomere lengths for the five TA sites (‘distal’, ‘proximal’, ‘middle’, ‘medial’, ‘lateral’) of individual tibialis anterior muscles for ankle positions of full dorsiflexion, intermediate, and full plantarflexion.

| **Landmark** | **Animal ID** | **Sarcomere length (µm)** | | | | | | | | | | | | | |
| --- | --- | --- | --- | --- | --- | --- | --- | --- | --- | --- | --- | --- | --- | --- | --- |
|  |  | **Full Dorsiflexion** | | | |  | **Intermediate angle** | | | |  | **Full Plantarflexion** | | | |
|  |  | **Mean** | **Std** | **Min** | **Max** |  | **Mean** | **Std** | **Min** | **Max** |  | **Mean** | **Std** | **Min** | **Max** |
| **Distal** | 1 | 2.14 | 0.08 | 1.91 | 2.38 |  | 2.37 | 0.09 | 2.09 | 2.62 |  | 2.63 | 0.10 | 2.25 | 3.02 |
|  | 2 | 2.20 | 0.08 | 1.85 | 2.43 |  | 2.63 | 0.06 | 2.32 | 2.82 |  | 2.72 | 0.09 | 2.43 | 2.99 |
|  | 3 | 2.17 | 0.11 | 1.82 | 2.46 |  | 2.48 | 0.07 | 2.25 | 2.72 |  | 2.68 | 0.06 | 2.51 | 2.83 |
|  | 4 | 2.18 | 0.09 | 1.94 | 2.45 |  | 2.63 | 0.10 | 2.25 | 2.95 |  | 2.77 | 0.08 | 2.55 | 3.04 |
|  | 5 | 2.27 | 0.10 | 1.90 | 2.52 |  | 2.62 | 0.08 | 2.36 | 2.87 |  | 2.70 | 0.09 | 2.49 | 3.03 |
| **Proximal** | 1 | 1.98 | 0.10 | 1.75 | 2.23 |  | 2.26 | 0.13 | 1.92 | 2.59 |  | 2.20 | 0.11 | 1.88 | 2.49 |
|  | 2 | 2.12 | 0.11 | 1.79 | 2.45 |  | 1.99 | 0.12 | 1.61 | 2.28 |  | 2.22 | 0.07 | 1.91 | 2.48 |
|  | 3 | 2.25 | 0.23 | 1.75 | 2.68 |  | 2.06 | 0.14 | 1.65 | 2.49 |  | 2.45 | 0.12 | 2.02 | 2.76 |
|  | 4 | 2.13 | 0.14 | 1.75 | 2.47 |  | 2.44 | 0.09 | 2.09 | 2.76 |  | 2.45 | 0.04 | 2.32 | 2.58 |
|  | 5 | 2.11 | 0.25 | 1.62 | 2.80 |  | 2.30 | 0.12 | 1.92 | 2.55 |  | 2.33 | 0.15 | 1.93 | 2.70 |
| **Middle** | 1 | 2.16 | 0.09 | 1.91 | 2.47 |  | 2.46 | 0.09 | 2.09 | 2.82 |  | 2.28 | 0.08 | 1.99 | 2.59 |
|  | 2 | 2.32 | 0.05 | 2.17 | 2.51 |  | 2.57 | 0.05 | 2.38 | 2.78 |  | 2.56 | 0.12 | 2.34 | 2.84 |
|  | 3 | 2.29 | 0.10 | 1.99 | 2.65 |  | 2.56 | 0.06 | 2.33 | 2.80 |  | 2.48 | 0.08 | 2.18 | 2.74 |
|  | 4 | 2.38 | 0.08 | 2.08 | 2.63 |  | 2.51 | 0.07 | 2.30 | 2.79 |  | 2.56 | 0.06 | 2.32 | 2.75 |
|  | 5 | 2.23 | 0.07 | 2.01 | 2.42 |  | 2.43 | 0.08 | 2.11 | 2.73 |  | 2.56 | 0.07 | 2.38 | 2.81 |
| **Medial** | 1 | 2.19 | 0.07 | 1.93 | 2.39 |  | 2.24 | 0.09 | 1.96 | 2.50 |  | 2.44 | 0.09 | 2.08 | 2.70 |
|  | 2 | 2.34 | 0.04 | 2.20 | 2.49 |  | 2.38 | 0.05 | 2.23 | 2.53 |  | 2.35 | 0.07 | 2.12 | 2.68 |
|  | 3 | 2.27 | 0.07 | 2.03 | 2.48 |  | 2.52 | 0.14 | 2.21 | 2.92 |  | 2.55 | 0.09 | 2.26 | 2.84 |
|  | 4 | 2.40 | 0.08 | 2.15 | 2.76 |  | 2.57 | 0.05 | 2.40 | 2.76 |  | 2.68 | 0.08 | 2.45 | 2.98 |
|  | 5 | 2.25 | 0.08 | 1.99 | 2.50 |  | 2.39 | 0.07 | 2.15 | 2.64 |  | 2.50 | 0.08 | 2.26 | 2.75 |
| **Lateral** | 1 | 2.18 | 0.08 | 1.96 | 2.48 |  | 2.36 | 0.08 | 2.09 | 2.61 |  | 2.35 | 0.08 | 2.05 | 2.61 |
|  | 2 | 2.39 | 0.05 | 2.20 | 2.58 |  | 2.55 | 0.06 | 2.32 | 2.73 |  | 2.49 | 0.05 | 2.33 | 2.67 |
|  | 3 | 2.20 | 0.08 | 1.97 | 2.42 |  | 2.45 | 0.08 | 2.17 | 2.70 |  | 2.63 | 0.07 | 2.43 | 2.91 |
|  | 4 | 2.36 | 0.07 | 2.12 | 2.67 |  | 2.57 | 0.07 | 2.35 | 2.84 |  | 2.67 | 0.09 | 2.37 | 2.99 |
|  | 5 | 2.20 | 0.08 | 1.92 | 2.44 |  | 2.50 | 0.07 | 2.29 | 2.71 |  | 2.86 | 0.10 | 2.57 | 3.22 |
|  |  |  |  |  |  |  |  |  |  |  |  |  |  |  |  |
|  |  | **Optimal lengths (2.30 - 2.47 µm)** | | | | | | | |  |  |  |  |  |  |
|  |  | **Ascending lengths (< 2.30 µm)** | | | |  |  |  |  |  |  |  |  |  |  |
|  |  | **Descending lengths (> 2.47 µm)** | | | | | |  |  |  |  |  |  |  |  |
